# Moral Evaluations of Organ Transplantation Influence Judgments of Death and Causation

**DOI:** 10.1007/s12152-015-9239-2

**Published:** 2015-08-11

**Authors:** Michael Nair-Collins, Mary A. Gerend

**Affiliations:** Behavioral Sciences and Social Medicine, Florida State University College of Medicine, 1115 West Call Street, Tallahassee, FL 32306 USA; Medical Social Sciences, Feinberg School of Medicine, Northwestern University, 633 N. St. Clair Street, Suite 1900, Chicago, IL 60611 USA

**Keywords:** Death, Brain death, Organ transplantation, Organ donation, Moral psychology

## Abstract

Two experiments investigated whether moral evaluations of organ transplantation influence judgments of death and causation. Participants’ beliefs about whether an unconscious organ donor was dead and whether organ removal caused death in a hypothetical vignette varied depending on the moral valence of the vignette. Those who were randomly assigned to the good condition (vs. bad) were more likely to believe that the donor was dead prior to organ removal and that organ removal did not cause death. Furthermore, attitudes toward euthanasia and organ donation independently predicted judgments of death and causation, regardless of experimental condition. The results are discussed in light of the framework of motivated reasoning, in which motivation influences the selection of cognitive processes and representations applied to a given domain, as well as Knobe’s person-as-moralist model, in which many basic concepts are appropriately imbued with moral features. On either explanatory framework, these data cast doubt on the psychological legitimacy of the mainstream justification for vital organ procurement from heart-beating donors, which holds that neurological criteria for death are scientifically justified, independently of concerns about organ transplantation. These data suggest that, rather than concluding that organ removal is permissible *because the donor is dead*, people may believe that the donor is dead *because they believe organ removal to be permissible*.

## Introduction

The practice of organ transplantation is premised on an ethical constraint known as the dead donor rule, which states that the removal of vital organs must not cause the death of the donor. This deontic constraint forbids the causing of one patient’s death in order to benefit others, even if the patient is unconscious, debilitated, or very near death [1]. It serves to protect vulnerable patients from exploitation, to maintain public trust in the transplantation enterprise, and has been described as “a centerpiece of the social order’s commitment to respect for persons and human life” ([1], p. 6). The majority of transplant organs are procured from patients who have been declared dead by neurologic criteria, or, “brain dead”, and thus the removal of organs from these patients is believed to accord with the dead donor rule. Furthermore, the prevailing view holds that from a biomedical perspective, neurological and circulatory criteria for the determination of death are simply two different ways of identifying the same underlying state of human death [2]. According to this view, neurological criteria for death are based on sound biomedical science and philosophical reasoning, and are justified independently of other concerns, including organ transplantation [3].

Although mostly accepted in the medical and legal communities [4], neurological criteria for death are controversial. Scholars have argued that brain death is insufficient for the death of a human organism in a biological sense, since patients who meet diagnostic criteria for brain death retain the capacity to function as an integrated whole in the maintenance of homeostasis and resistance of entropy [5, 6]. Furthermore, some argue that rather than reflecting an advance in the scientific understanding of death, the concept of brain death embodies or reflects an implicit moral evaluation, specifically, that it is permissible to remove vital organs in the condition of devastating neurological injury known as brain death [7, 8]. Thus, rather than concluding that organ removal is permissible *because the donor is dead*, perhaps people believe that the donor is dead *because they believe organ removal to be permissible*.

In this article, we describe two experiments designed to ascertain whether people’s moral evaluations of organ transplantation influence their judgments of death and causation in an organ removal scenario. We situate the results within the framework of motivated reasoning, in which motivation influences the selection of cognitive representations applied to a given domain. In the discussion section, we also consider an alternative theoretical framework for interpreting the results, similar to Knobe’s person-as-moralist model [9], in which the concepts of death and causation are treated as partially moral concepts, rather than value-neutral factual concepts (cf. [7]). Finally, we discuss the implications of these results for the bioethical debate on death and organ transplantation.

### Motivated Reasoning and Organ Transplantation

Motivated reasoning is an empirically well-validated framework that explains people’s reasoning processes in terms of an interaction of motivational and cognitive processes. Motivation – that is, any wish, desire, or preference to reach a particular conclusion – impacts the reasoning process by influencing the selection of cognitive processes and representations applied to a given reasoning problem in such a way that the outcome of the reasoning process is biased towards the preferred conclusion [10]. Motivated reasoning has been found in a variety of domains, including beliefs about the self and others [10, 11], and in moral cognition [12].

Particularly relevant to the present context, there are a series of findings of motivated reasoning involving blame, causation, and harm. Alicke [13, 14] found that people tend to interpret relevant facts about a given situation in a way that validates a prior assessment of blame. For example, people were more likely to blame the driver of a car for being involved in an accident when he was speeding home to hide a vial of cocaine from his parents, than when he was speeding home to hide an anniversary present. In accordance with motivated reasoning, people were also more likely to judge the driver as the *cause* of the accident when he was speeding home to hide cocaine (vs. an anniversary present), thus validating their prior assessment of blame, even though all other factors were the same between the two cases [13]. In a legal context, Sood and Darley [15] found motivated reasoning about the concept of harm. Participants were more likely to assert that harm is caused by some distasteful action (such as washing a sidewalk with the flag) when they believed that the law requires showing the occurrence of harm in order to criminalize the behavior they found distasteful. However, when not constrained by demonstrable harm as a requirement of criminalization, participants who found the action distasteful and wished to see it criminalized did not assert that it caused harm, thus demonstrating that judgments of harm were recruited in a motivated fashion in order to justify their preference for criminalization. Similarly, Miller et al. [16] have argued that much of what is taken as received wisdom in medical ethics relies on a series of moral fictions [16] or moral bias [5], which involve the motivated misinterpretation of facts about causation and intention in order to allow accepted medical practices at the end of life, such as withdrawing life-sustaining treatment, to appear consistent with traditional norms, such as that doctors must not cause death. Although plausible, they do not present experimental evidence for motivated reasoning in this specific context.

The theoretical framework of motivated reasoning is useful for investigating beliefs about death and organ transplantation. If an observer were to judge organ removal from a given donor as morally good or as not blameworthy, then according to the motivated reasoning model, that observer would be motivated to interpret the scenario in a way that validates the observer’s ethical judgment, for example, by concluding that the act of removing organs did not cause death, and that the donor was already dead before organs were removed. On the other hand, were an observer to judge organ removal as morally bad or as blameworthy, then this would create a motivation to interpret the scenario differently, by concluding that the donor was still alive when organs were removed, and that organ removal was the cause of death.

In light of this background, we predicted that people’s beliefs about whether an unconscious organ donor was dead and whether organ removal caused death in a hypothetical vignette would vary depending on whether the vignette was framed as morally good or bad. Participants presented with the good scenario would be more likely to judge that the unconscious donor was already dead prior to organ removal and that organ removal did not cause death, as compared to the bad scenario. Further, we expected that individual differences in attitudes toward organ transplantation and euthanasia would predict participants’ beliefs about whether the organ donor was dead, and whether organ removal caused death, regardless of experimental condition. That is, participants with more favorable attitudes toward organ donation in general would be more likely to believe that the donor was dead and organ removal did not cause death than those with less favorable attitudes, regardless of whether they read the morally good or bad scenario. Conversely, participants with less favorable attitudes toward euthanasia would be more likely to believe that the donor was alive and that organ removal caused death than those with more favorable attitudes.

## Study 1

### Method

#### Participants and Recruitment

Participants were recruited through Amazon Mechanical Turk, an online crowdsourcing platform that has been successfully used in experimental psychological research [17, 18]. Recruitment was limited to individuals 18 years of age or older who resided in the United States. To estimate the necessary sample size we conducted a power analysis in which we expected a moderate effect of experimental condition and small effects of the attitudes scales (described below). A sample size of 223 was associated with 80 % power to detect the hypothesized effects. To account for participants who would be excluded (e.g., incomplete responses and attention check failures), an additional 50 participants were allowed to participate in the study.

A total of 273 participants enrolled in the study. The final sample size was reduced to 219 after removing participants with incomplete responses (e.g., completed the first question only; *n* = 22), participants who failed at least one of four attention checks (e.g., failed to type a specific phrase; *n* = 17), and participants who completed the study more than once as indicated by a duplicate IP address (*n* = 15). If an individual completed the study more than once we included the first completed set of results in the database. Mean age was 38 years (*SD* = 14) and 61 % of participants were female.

In addition to standard demographics, we collected information on potential confounds such as professional training, experience with brain death or organ transplantation, and religious beliefs. See Table 1 for details on demographics and background characteristics.

**Table 1:** Participant Demographics, Background Characteristics and Attitudes for Study 1 and 2

Variable	Study 1	Study 2
	Mean (SD) or N (%)	Mean (SD) or N (%)
Demographics		
Age (years)	38.4 (14.1)	33.1 (11.4)
Gender		
Male	85 (39)	136 (51)
Female	133 (61)	129 (48)
Transgender	1 (<1)	2 (<1)
English native language		
Yes	216 (99)	262 (99)
No	2 (1)	4 (2)
Race/Ethnicity		
White, non-Hispanic	177 (81)	201 (75)
Black, non-Hispanic	10 (5)	21 (8)
Hispanic	12 (6)	18 (7)
American Indian/Alaskan Native	1 (<1)	1 (<1)
Asian/Pacific Islander	12 (6)	16 (6)
Multiracial	7 (3)	10 (4)
Education		
Elementary	0 (0)	1 (<1)
Some high school	3 (1)	1 (<1)
High school graduate or GED	32 (15)	22 (8)
Some college or technical school	79 (36)	119 (45)
College graduate	104 (48)	124 (46)
Religious affiliation		
Buddhist	2 (1)	9 (3)
Christian (All)	107 (49)	118 (44)
Hindu	1 (<1)	0 (0)
Jewish	5 (2)	3 (1)
Muslim	3 (1)	4 (1)
None	85 (39)	118 (44)
Other	16 (7)	15 (6)
How religious^a^	4.2 (3.3)	3.7 (3.2)
Background characteristics^b^		
Know someone who donated an organ while living	41 (19)	52 (20)
Know someone who donated an organ after death	74 (34)	60 (23)
Know someone who has received an organ transplant	80 (37)	74 (28)
Received education about organ donation	130 (59)	142 (53)
Personally seen a person who was diagnosed as “brain dead” or “dead by neurologic criteria”	49 (22)	48 (18)
Health professional	11 (5)	19 (7)
*Attitudes*		
Attitudes toward organ donation^c^	60.3 (8.6)	60.0 (8.6)
Attitudes toward euthanasia^d^	30.3 (9.3)	30.6 (8.9)

Percentages may not add up to 100 due to rounding error

^a^ Participants selected a number from 1 (not religious) to 10 (very religious) in response to the statement: “I view myself as…”

^b^ Number (percentage) responding “yes” to each statement

^c^ Scores range from 18–72 with higher scores indicating a more favorable attitude toward organ donation

^d^ Scores range from 10–50 with higher scores indicating a more favorable attitude toward euthanasia

#### Procedures and Materials

The study was approved by the Florida State University Human Subjects Committee. After providing informed consent, participants completed an approximately 10-min online survey for which they received a small payment. To account for possible order effects, the study used a 2 (moral valence: good vs. bad) X 2 (order: read vignette first vs. completed attitudes scales first) between-subjects factorial design. Participants were randomly assigned to read a vignette about an organ transplant scenario that was framed to occur under circumstances that differed in moral valence (details below). Participants completed all outcome measures immediately after reading the vignette regardless of order. Instructions made clear that there are no right or wrong answers for these questions and that we were interested in participants’ personal opinions. Finally, participants answered demographic and background questions. At the end of the study, we emphasized to participants that the vignettes were fictional.

##### Vignette

The aim of the vignette was to describe a patient in an irreversible apneic coma (using lay terms) who might be a candidate for organ donation, though without using potentially confounding terms such as “death” or “brain death”. Furthermore, the two conditions were constructed to be the same in every relevant way except for the experimental manipulation of moral valence. While we have labeled the conditions as “good” and “bad”, the key aspect of the design is the *contrast* between the two conditions, with the bad condition being of more obviously negative moral valence than the good condition. If our hypothesis is correct, we would expect that the difference in moral valence between the conditions would influence participants’ judgments of death and causation.

The scenario began the same way for all participants, where they read about a man named John who had been severely injured in a car accident.

John has been in a very bad car accident. He suffered a severe head injury and is now in the hospital. As a result of the injury, John is completely unconscious. He cannot hear or feel anything, cannot remember or think about anything, and his condition is irreversible. John will never wake up. He also cannot breathe without mechanical support, but he is on a breathing machine that keeps his lungs working. Without the machine, John’s heart and all of his other organs would stop within minutes.

Then participants read about the circumstances under which John’s organs had been removed. Approximately half of participants read the morally good scenario and half read the morally bad scenario.

[Good condition:] John has a signed organ donor card, and his family has agreed to organ donation. They know that he wanted to donate his organs, and they believe that by giving the gift of life and saving up to 6 other people’s lives with his organs, at least they can find some meaning from this senseless tragedy. With the family’s permission, the hospital staff take John to the operating room while he is still on the breathing machine, and the surgeon removes John’s liver, pancreas, both kidneys, heart, and lungs, and transplants them into other patients.

[Bad condition:] John has a signed living will stating that he does not want to donate any organs for religious reasons, and his family, who share his religion, are opposed to organ donation. They know that he did not want to donate his organs, and they believe that by violating his wishes they would only make this senseless tragedy worse. The transplant surgeon knows that John and his family have refused organ donation, but thinks to himself, “I don’t care at all what this bum wanted or what his family wants. I hate them because of their race, and because of their religion. I’m going to take his organs because it will let them know that they are not welcome in this hospital.” In the middle of the night when his family went home to get some much needed rest, the hospital staff take John to the operating room while he is still on the breathing machine, and the surgeon removes John’s liver, pancreas, both kidneys, heart, and lungs, and transplants them into other patients.

#### Measures

##### Outcome Measures

Immediately after reading the vignette participants were asked to rate their agreement with the following statements using a 5-point Likert scale (1 = strongly disagree; 2 = disagree; 3 = unsure; 4 = agree; 5 = strongly agree): “John was already dead before his organs were removed.” “The surgeon caused John’s death.” “The surgeon is responsible for John’s death.” Afterward, participants rated the same three items using a forced-choice “yes” or “no” scale with the following instructions: “If you had to choose just one, would you say that: John was already dead…” Responses of yes were coded as 1 and responses of no were coded as 0. Finally, participants were asked “What would be the best description of how John died?” and selected one response from the following choices: 1 = The car accident was the only cause of John’s death; 2 = The car accident was mostly the cause of John’s death, though the surgeon contributed by removing John’s organs; 3 = The car accident and the surgeon played an equal role in John’s death; 4 = The surgeon’s removal of John’s organs was mostly the cause of John’s death, though the car accident contributed; 5 = The surgeon was the only cause of John’s death.

##### Attitudes Toward Organ Donation

The Organ Donation Attitude Scale (ODAS) [19] is an 18-item validated scale for assessing attitudes toward organ donation. Participants indicate their agreement with each statement on a 4-point Likert scale (1 = strongly disagree to 4 = strongly agree). Sample statements include: “I support organ donation” and “I believe that organ donation is against my religion” (reverse scored). Items are summed to create a scale score that can range from 18–72, with higher scores indicating more positive attitudes toward organ donation. Coefficient alpha for the ODAS was .92. (See Table 1 for descriptive statistics.)

##### Attitudes Toward Euthanasia

The Attitudes Toward Euthanasia scale (ATE) [20] is a 10-item validated scale for assessing attitudes toward euthanasia. Participants indicate their agreement with each statement on a 5-point Likert scale (1 = strongly disagree to 5 = strongly agree, where 3 = undecided). Of note, none of the statements use the word “euthanasia.” The conceptual dimensions reflected in the scale include the active/passive distinction (i.e., “killing vs. letting die”), the reason for the termination of life (severe pain, no possibility of recovery), and locus of decision-making (patient request, doctor’s decision). Sample statements include: “It is okay for a doctor to administer enough medicine to end a patient’s life if the doctor does not believe that they will recover,” and “It is okay for a doctor to remove a patient’s life-support and let them die if the doctor thinks that the patient’s pain is too severe.” Items are summed to create a scale score that can range from 10–50, with higher scores indicating more positive attitudes toward euthanasia. Coefficient alpha for the ATE scale was .92. (See Table 1 for descriptive statistics.)

### Results

#### Randomization Checks

To assess whether randomization was successful, we used *t*-tests and chi-square analyses to compare participants in the morally good vs. bad condition on demographics, background characteristics, and attitudes. With one exception, participants in the two conditions were not significantly different from each other in regard to demographic variables, background characteristics, or attitudes (data not shown). A higher percentage of participants in the good condition (41 %) reported knowing someone who had donated an organ after death relative to participants in the bad condition (27 %), χ^2^ (1, *N* = 219) = 5.01, *p* = .032. However, this difference was no longer statistically significant after adjusting for multiple comparisons (Bonferroni correction, *p* = .05/16 = .003).

#### Effect of Condition, Order, and Their Interaction on Outcome Measures

We used analysis of covariance (ANCOVA) to examine effects of experimental condition (good vs. bad), order (vignette first vs. attitude scales first), and the condition by order interaction on the Likert-rating outcome variables, controlling for attitudes toward organ donation and attitudes toward euthanasia. (Effects of the attitude variables are reported further below.) After centering all predictor variables [21], we used logistic regression to examine effects of condition, order, and the condition by order interaction on the forced-choice (yes/no) outcome variables, again controlling for the two attitudes scales. In these analyses, the bad condition served as the reference group. All statistical tests were two-tailed.

##### John was Already Dead

We observed a significant effect of experimental condition on participants’ Likert rating of whether John was already dead before his organs were removed. (See Table 2 for means and percentages of outcome variables for participants in the morally good vs. bad condition.) Relative to participants in the bad condition, participants in the good condition were more likely to agree that John was already dead before his organs were removed, *F* (1, 204) = 45.37, *p* < .001, partial η^2^ = .18. No order effects emerged and the condition by order interaction was non-significant. Likewise, a significant effect of condition was observed for the forced-choice version of this item, Wald = 27.00, *p* < .001, OR = 5.35, 95 % CI [2.84, 10.08]. Participants in the good condition were over 5 times more likely to state that the patient was already dead.

**Table 2:** Means and Standard Deviations for Outcome Variables (Study 1 and 2)

Primary outcome variables Likert-type	Study 1		Study 2	
	Good	Bad	Good	Bad
	Mean (SD) *n* = 110	Mean (SD) *n* = 109	Mean (SD) *n* = 127	Mean (SD) *n* = 140
John was already dead before his organs were removed^a^	3.6 (1.3)	2.4 (1.2)	2.9 (1.3)	2.4 (1.3)
The surgeon caused John’s death.^a^	2.1 (1.2)	3.4 (1.3)	2.6 (1.3)	3.6 (1.3)
The surgeon is responsible for John’s death.^a^	1.9 (1.1)	3.5 (1.4)	2.5 (1.3)	3.6 (1.4)
Best description for how John died.^b^	1.6 (1.0)	2.7 (1.2)	1.9 (1.2)	2.6 (1.3)
Primary outcome variables Forced-choice rating^c^	Study 1		Study 2	
	Good N (%) *n* = 110	Bad N (%) *n* = 109	Good N (%) *n* = 127	Bad N (%) *n* = 140
John was already dead before his organs were removed.	75 (69)	37 (34)	68 (54)	49 (35)
The surgeon caused John’s death.	22 (20)	67 (62)	42 (33)	91 (66)
The surgeon is responsible for John’s death.	15 (14)	69 (64)	42 (33)	92 (66)

“John” is replaced with “the patient” for items in Study 2

^a^ Rated on a 5-point Likert scale (1 = strongly disagree; 2 = disagree; 3 = unsure; 4 = agree; 5 = strongly agree)

^b^ Rated on a 5-point scale (1 = The car accident was the only cause of John’s death; 2 = The car accident was mostly the cause of John’s death, though the surgeon contributed by removing John’s organs; 3 = The car accident and the surgeon played an equal role in John’s death; 4 = The surgeon’s removal of John’s organs was mostly the cause of John’s death, though the car accident contributed; 5 = The surgeon was the only cause of John’s death.)

^c^ Number (percentage) responding “yes” to each statement

##### The Surgeon Caused John’s Death

We observed a significant effect of condition on participants’ perceptions of whether the surgeon caused John’s death. Participants in the good (vs. bad) condition were less likely to agree that the surgeon caused John’s death, *F* (1, 204) = 58.29, *p* < .001, partial η^2^ = .22. No order or interaction effects emerged. This effect of condition was also observed for the forced-choice version of this item, Wald = 34.31, *p* < .001, OR = 0.14, 95 % CI [0.07, 0.27]. Participants in the good condition were 86 % less likely to state that the surgeon caused John’s death.

##### The Surgeon is Responsible for John’s Death

A similar pattern was observed for participants’ perceptions of whether the surgeon is responsible for John’s death. Participants in the good (vs. bad) condition were less likely to agree that the surgeon was responsible for John’s death, *F* (1, 204) = 86.19, *p* < .001, partial η^2^ = .30. No order or interaction effects emerged. This effect of condition was also observed the forced-choice version of this item, Wald = 44.94, *p* < .001, OR = 0.08, 95 % CI [0.04, 0.16]. Participants in the good condition were 92 % less likely to state that the surgeon was responsible for John’s death.

##### Best Description of John’s Death

We observed a significant effect of condition on participants’ ratings of the best description of John’s death, *F* (1, 204) = 48.94, *p* < .001, partial η^2^ = .19. Participants in the morally good condition largely endorsed the car accident as the sole cause of John’s death whereas participants in the bad condition attributed John’s death to both the car accident and the surgeon. No order effects emerged and the condition by order interaction was non-significant.

#### Effects of Attitudes toward Organ Donation and Euthanasia on Outcome Measures

Attitudes toward organ donation and euthanasia were included in all of the models described above to assess whether these variables predicted responses to the outcome variables over and above the effect of experimental condition. A small positive correlation was observed between the ODAS and ATE, *r* (208) = .15, *p* = .028.

##### John was Already Dead

We observed a significant effect of attitudes toward euthanasia such that participants who held less favorable attitudes toward euthanasia were less likely to agree that John was dead prior to the organ removal surgery, *F* (1, 204) = 8.40, *p* = .004, partial η^2^ = .04. A similar effect was observed for the forced-choice version of the item, Wald = 12.16, *p* < .001, OR = 1.07, 95 % CI [1.03, 1.11]. Attitudes toward organ donation showed a similar, though less strong pattern. Participants who held more favorable attitudes toward organ donation were more likely to agree that John was already dead prior to organ removal, *F* (1, 204) = 10.74, *p* = .001, partial η^2^ = .05. A similar effect was observed for the forced-choice version of this item that approached but did not reach statistical significance, Wald = 2.94, *p* = .087, OR = 1.03, 95 % CI [1.00, 1.07].

##### The Surgeon Caused John’s Death

We observed a significant effect of attitudes toward euthanasia such that participants who had less favorable attitudes toward euthanasia were more likely to judge that the surgeon caused John’s death, *F* (1, 204) = 8.09, *p* = .005, partial η^2^ = .04. A significant effect was also observed for the forced-choice version of this item, Wald = 6.72, *p* = .01, OR = 0.95, 95 % CI [0.92, 0.99]. Attitudes toward organ donation also demonstrated the expected pattern, in which participants who held more favorable attitudes toward organ donation were less likely to agree that the surgeon caused John’s death, *F* (1, 204) = 6.74, *p* = .01, partial η^2^ = .03. A similar pattern was observed for the forced-choice version of this item, Wald = 4.51, *p* = .034, OR = 0.96, 95 % CI [0.92, 1.00].

##### The Surgeon is Responsible for John’s Death

A significant effect of attitudes toward euthanasia was observed, such that participants who held less favorable attitudes were more likely to agree that the surgeon is responsible for John’s death, *F* (1, 204) = 6.45, *p* = .012, partial η^2^ = .03. Likewise, a significant effect of attitudes toward euthanasia was observed for the forced-choice version of this item, Wald = 4.55, *p* = .033, OR = 0.96, 95 % CI [0.92, 1.00]. A similar though less strong pattern was observed for attitudes toward organ donation. Participants who were more favorable to organ donation were less likely to agree that the surgeon is responsible for John’s death, *F* (1, 204) = 6.16, *p* = .014, partial η^2^ = .03. A similar effect was observed for the forced-choice version of this item that approached but did not reach statistical significance, Wald = 3.63, *p* = .057, OR = 0.96, 95 % CI [0.92, 1.00].

##### Best Description of John’s Death

We observed a significant effect of attitudes toward euthanasia whereby participants who held less favorable attitudes toward euthanasia were more likely to attribute the cause of John’s death to both the surgeon and the car accident (vs. the car accident alone), *F* (1, 204) = 11.00, *p* = .001, partial η^2^ = .05. Attitudes toward organ donation, however, was not a statistically significant predictor of the best description of John’s death, *F* (1, 204) = .08, *p* = .776, partial η^2^ = .00.

### Discussion

We found that people’s beliefs about whether an unconscious organ donor was dead and whether organ removal caused death in a hypothetical vignette varied depending on the moral valence of the vignette. Participants’ judgments about death and causation in this scenario were assessed in a number of ways including a Likert-type scale, a forced-choice response set, and a response set that enabled participants to attribute partial causation to both the surgeon and the car accident. In every case we found the predicted effect. Further, individual differences in participants’ attitudes toward organ donation and toward euthanasia, in general, predicted judgments about death and causation regardless of experimental condition.

A few exceptions were noted in which attitudes toward organ donation did not predict some of the outcome variables. One possible explanation is that attitudes toward organ donation create a weaker motivation to reach conclusions about death and causation with respect to a particular scenario, than do moral evaluations specifically of that same scenario. For example, when participants read the morally bad scenario, this likely triggered an immediate negative moral evaluation of the actors involved, creating the motivation to reach blame-validating conclusions about death and causation, which then biased cognitive processes toward reaching the preferred conclusions. Although general attitudes toward organ transplantation can trigger the same process, it is possible that their effects are less strong. However, given that attitudes toward euthanasia were statistically significant predictors of judgments of death and causation for all outcome variables, a second possibility is that the construct validity of the ODAS could be improved.

## Study 2

The goal of Study 2 was to replicate findings from Study 1 using a different sample of participants and a different scenario.

### Method

#### Participants

Participants were recruited through Mechanical Turk. Based on the results of Study 1 we determined that a sample size of 223 would have 80 % power to detect the hypothesized effects in Study 2. As in Study 1, we allowed additional participants to complete the study to account for attrition. Although 320 participants responded to the study, the final sample size was reduced to 267 after removing participants with incomplete responses (*n* = 18), participants who failed at least one of the four attention checks (*n* = 17), participants who completed the study more than once (*n* = 2), and participants who completed Study 1 (as derived from IP addresses; *n* = 12). Mean age was 33 years (*SD* =11) and 48 % of participants were female. See Table 1 for additional demographic and background characteristics.

#### Procedures, Materials, and Measures

The procedure for Study 2 was identical to Study 1 and used the same 2 (moral valence) X 2 (order) between-subjects design. The primary difference between the studies pertained to the details of the vignette.

##### Vignette

The aim of the vignette in Study 2 was similar to that in Study 1, except we changed potentially relevant variables, such as whether the patient had a name, the presence of a family, the role of religious beliefs, and the surgeon’s motive in the bad condition. Because little information was provided about the patient in Study 2’s vignette, the moral valence in the good condition was presumably more ambiguous. This allows for a stronger test of the hypothesis that moral evaluations of organ transplantation influence judgments of death and causation because the difference in moral valence between the good and bad conditions in Study 2 was presumably less pronounced than it was in Study 1.

The scenario began the same way for all participants:

A homeless man has been struck by a fast-moving car. He suffered a severe head injury and is now in the hospital. As a result of the injury, the man is completely unconscious. He cannot hear or feel anything, cannot remember or think about anything, and his condition is irreversible. This man will never wake up. He also cannot breathe without mechanical support, but is on a breathing machine that keeps his lungs working. Without the machine, the man’s heart and all of his other organs would stop within minutes. No family, contacts, or identification can be found for this man, so the hospital asked a judge to appoint a guardian who can make decisions on his behalf.

Then participants read about the circumstances under which the man’s organs had been removed. Approximately half of participants read the morally good scenario and half read the morally bad scenario.

[Good condition:] Dr. Jones is the transplant surgeon on duty. Knowing that this patient is a candidate to donate organs, Dr. Jones discusses this possibility with the guardian. They both agree that by giving the gift of life and by saving the lives of up to 6 other people through organ donation, at least some good can come from this senseless tragedy.

With the guardian’s permission, the hospital staff take the patient to the operating room while he is still on the breathing machine, and Dr. Jones removes the patient’s liver, pancreas, both kidneys, heart, and lungs, and transplants them into other patients.

[Bad condition:] Dr. Jones is the transplant surgeon on duty. Although he wants to remove the man’s organs, the court-appointed guardian has denied permission to do so. Dr. Jones thinks to himself, “This bum has contributed nothing to society. And now he’s here taking up a hospital bed, soaking up my valuable time and resources, which he does not deserve. Well, I don’t care what that guardian says. I’m going to remove his organs, because if I can get one more heart this month, I’m going to get a huge bonus and I’ll buy another boat”.

In the middle of the night when the guardian has left, the hospital staff take the patient to the operating room while he is still on the breathing machine, and Dr. Jones removes the patient’s liver, pancreas, both kidneys, heart, and lungs, and transplants them into other patients.

Participants completed the same attitudes scales, demographic and background characteristics, and attention checks as in Study 1. As in Study 1, we emphasized that there are no right or wrong answers and we are interested in participants’ personal opinions. We also stated that the vignettes were fictional at the end of the study. Coefficient alpha was computed for the ODAS (α = .91) and ATE (α = .90) scales. The outcome variables were identical to those in the first study except that “the patient” was substituted for the name “John.” The analysis strategy was identical to Study 1.

### Results

#### Randomization Checks

Participants in the morally good vs. bad condition were not significantly different from each other in regards to demographic variables, background characteristics, or attitudes (data not shown).

#### Effect of Condition, Order, and their Interaction on Outcome Measures

##### The Patient was Already Dead

As in Study 1, we observed a significant effect of experimental condition on participants’ Likert rating of whether the patient was already dead before his organs were removed. (See Table 2 for means and percentages of outcome variables for participants in the morally good vs. bad condition.) Participants in the good condition (vs. bad) were more likely to agree that the patient was already dead before his organs were removed, *F* (1, 255) = 10.81, *p* = .001, partial η^2^ = .04. No order effects emerged and the condition by order interaction was non-significant. A significant effect of condition was also observed for the forced-choice version of this item, Wald = 11.04, *p* < .001, OR = 2.43, 95 % CI [1.44, 4.11]. Participants in the good condition were over 2 times more likely to state that the patient was already dead.

##### The Surgeon Caused the Patient’s Death

We observed a significant effect of condition on participants’ judgments of whether the surgeon caused the patient’s death, as in Study 1. Relative to those in the bad condition, participants in the good condition were less likely to agree that the surgeon caused death, *F* (1, 255) = 37.47, *p* < .001, partial η^2^ = .13. No order or interaction effects emerged. This effect was also observed for the forced-choice version of this item, Wald = 28.16, *p* < .001, OR = 0.23, 95 % CI [0.14, 0.40]. Participants in the good condition were 77 % less likely to state that the surgeon caused John’s death.

##### The Surgeon is Responsible for the Patient’s Death

A similar pattern was observed for participants’ perceptions of whether the surgeon is responsible for the patient’s death. Participants in the morally good (vs. bad) condition were less likely to agree that the surgeon was responsible for death, *F* (1, 254) = 49.40, *p* < .001, partial η^2^ = .16. No order or interaction effects emerged. This effect of condition was observed the forced-choice version of this item as well, Wald = 28.69, *p* < .001, OR = 0.23, 95 % CI [0.13, 0.39]. Participants in the good condition were 77 % less likely to state that the surgeon was responsible for John’s death.

##### Best Description of the Patient’s Death

As in Study 1, we observed a significant effect of condition on participants’ ratings of the best description of the patient’s death, *F* (1, 255) = 22.82, *p* < .001, partial η^2^ = .08. While participants in the morally good condition largely endorsed the car accident as the sole cause of death, those in the bad condition attributed the patient’s death to both the car accident and the surgeon. No order effects emerged and the condition by order interaction was non-significant.

#### Effects of Attitudes Toward Organ Donation and Euthanasia on Outcome Measures

As in Study 1, attitudes toward organ donation and euthanasia were included in all of the models to assess whether these variables predicted responses to the outcome variables over and above the effect of experimental condition. A small positive correlation was again observed between the ODAS and ATE, *r* (259) = .22, *p* < .001.

##### The Patient was Already Dead

Similar to Study 1, we observed a significant effect of attitudes toward euthanasia such that participants who held less favorable attitudes toward euthanasia were less likely to agree that the patient was dead prior to the organ removal surgery, *F* (1, 255) = 15.14, *p* < .001, partial η^2^ = .06. A similar effect was observed for the forced-choice version of the item, Wald = 10.64, *p* = .001, OR = 1.05, 95 % CI [1.02, 1.09]. As in Study 1, attitudes toward organ donation showed a less consistent pattern. Attitudes toward organ donation were not significant predictors of agreement that the patient was dead prior to organ removal for the Likert version of this item, *F* (1, 255) = 2.11, *p* = .148, partial η^2^ = .01. However, we observed a significant effect of attitudes toward organ donation for the forced-choice version, whereby participants who held more favorable attitudes toward organ donation were more likely to agree that the patient was already dead, Wald = 3.91, *p* = .048, OR = 1.03, 95 % CI [1.00, 1.07].

##### The Surgeon Caused the Patient’s Death

The expected pattern was observed for attitudes toward euthanasia such that participants who had less favorable attitudes were more likely to judge that the surgeon caused the patient’s death, *F* (1, 255) = 9.33, *p* = .002, partial η^2^ = .04. Likewise, a significant effect was observed for the forced-choice version of this item, Wald = 4.99, *p* = .026, OR = 0.97, 95 % CI [0.94, 1.00]. A similar though less strong pattern was observed for attitudes toward organ donation, in which participants who held more favorable attitudes toward organ donation were less likely to agree that the surgeon caused death, though this effect did not reach statistical significance, *F* (1, 255) = 2.57, *p* = .110, partial η^2^ = .01. A significant effect was observed for the forced-choice version of this item, Wald = 4.15, *p* = .042, OR = 0.97, 95 % CI [0.94, 1.00].

##### The Surgeon is Responsible for the Patient’s Death

A significant effect of attitudes toward euthanasia was observed whereby participants who held less favorable attitudes were more likely to agree that the surgeon is responsible for the patient’s death, *F* (1, 254) = 9.68, *p* = .002, partial η^2^ = .04. The expected effect was also observed for the forced-choice version of this item, Wald = 6.46, *p* = .011, OR = 0.96, 95 % CI [0.93, 0.99]. Participants who held more favorable attitudes toward organ donation were less likely to agree that the surgeon is responsible for the patient’s death in the Likert version of this item, though this effect did not reach statistical significance *F* (1, 254) = 3.47, *p* = .064, partial η^2^ = .01. However, a significant effect was observed for the forced-choice version of this item, Wald = 4.75, *p* = .029, OR = 0.96, 95 % CI [0.93, 1.00].

##### Best Description of the Patient’s Death

Finally, we observed a significant effect of attitudes toward euthanasia whereby participants who held less favorable attitudes toward euthanasia were more likely to attribute the cause of the patient’s death to both the surgeon and the car accident (vs. the car accident alone), *F* (1, 255) = 7.34, *p* = .007, partial η^2^ = .03. We also observed a significant effect of attitudes toward organ donation, whereby participants who held more favorable attitudes toward organ donation were more likely to attribute the cause of the patient’s death to the car accident alone (vs. both the car accident and the surgeon), *F* (1, 255) = 9.89, *p* = .002, partial η^2^ = .04.

### Discussion

The findings of Study 1 were replicated in Study 2. Importantly, in Study 2 we changed several salient characteristics of the vignette, including the role of religious beliefs, the presence of a family, whether the patient had a name, and the surgeon’s motive in the bad condition, which was to gain a bonus rather than prejudice. This replication with a different sample and different vignettes provides further evidence for the role of moral evaluations in people’s judgments of death and causation.

## General Discussion

People randomly assigned to read a story about organ removal from a patient in irreversible apneic coma that was framed in a morally good (vs. bad) manner were more likely to say that the donor was dead and that surgery did not cause death, even though the physiologic state of the donor and physical circumstances of organ removal were exactly the same in both the good and bad versions of the story. Second, individual differences in attitudes toward organ donation and toward euthanasia both independently predicted people’s beliefs about death and causation in an organ removal scenario, regardless of experimental condition. Thus, the results of the experimental manipulation of moral valence, and the associations between the attitude scales and judgments of death and causation (regardless of experimental condition) mutually reinforce each other. These results were replicated in a second sample of participants, using different vignettes in which the contrast in moral valence between the good and bad conditions was less pronounced. Taken together, our findings support our hypothesis that moral evaluations of organ transplantation affect judgments of death and causation.

Although we have posed our hypotheses and interpreted our results in light of the motivated reasoning framework, these results are also relevant to a competing theoretical framework, Knobe’s *person-as-moralist* model [9]. We will address both the motivated reasoning and the person-as-moralist models below as potential explanations of these results, and then discuss their relevance to bioethical debates about death and organ transplantation.

### Death and Motivated Reasoning

Our findings can be conceptualized in terms of motivated reasoning, in which any preference, wish, or desire to reach a particular conclusion impacts cognition in a way that biases the observer toward reaching the preferred conclusion. Participants differed in their general attitudes toward organ transplantation and euthanasia, as reflected in scores on the ODAS and ATE scales. Their favorable or unfavorable opinions toward these practices created the motivation to interpret relevant aspects of the vignette in a way that would validate their prior attitudes. Specifically, those who were more favorable to organ transplantation were more likely to believe that the comatose patient was already dead and that organ removal was not the cause of death, while those who were less favorable to euthanasia were motivated to reach the opposite conclusions. Similarly, the experimental manipulation of moral valence likely triggered spontaneous moral evaluations of organ removal in that specific scenario. Those participants who were randomized to read the morally bad versions of the stories likely judged the surgeon’s actions to be morally blameworthy, reflected in their higher agreement that the surgeon is responsible for the patient’s death (Table 2). This moral assessment created the motivation to interpret the scenario in a way that would validate that assessment, specifically by concluding that the patient was alive prior to organ removal and that the surgeon caused the patient’s death by removing organs. A parallel process occurred in the good condition, where participants were motivated to reach different conclusions about death and causation that would validate their different moral evaluations.

The effect sizes across experimental condition in Study 1 were consistently larger than those in Study 2 (reported above; see also Table 2 to compare means and percentages across conditions). This is consistent with and predicted by the model of motivated reasoning. In Study 1, the good condition was presumably more obviously good than in Study 2, because the patient wanted to be a donor and the family consented to donation, whereas in Study 2 the good condition was presumably more ambiguous, because there was no information about the donor’s prior wishes. Thus, in the good condition of Study 1 there was a stronger positive moral evaluation that organ removal was permissible (compared to the good condition of Study 2), creating a stronger motivation to reach conclusions about death and causation that would validate that moral evaluation. This stronger motivation in Study 1 (vs. Study 2), in turn, resulted in a larger difference in judgments of death and causation across condition, reflected in larger effect sizes in Study 1. In other words, the greater contrast in moral valence between the two conditions in Study 1 (vs. Study 2) yielded a greater contrast in motivations to interpret the scenario differently in the two conditions, which in turn is reflected in the larger differences in judgments of death and causation, and hence the larger effect sizes in Study 1.

Finally, blame-validation is a particular manifestation of the more general phenomenon of motivated cognition. In the specific experimental contexts reported here, it is likely that participants were motivated to blame an agent, thus influencing the outcome of judgments of death and causation. However, it is also plausible that the phenomenon under investigation is not specifically tied to blame-validation, but is part of a more general pattern of motivated cognition in which people (mis) interpret a variety of relevant facts about death, causation, and intention so as to allow desired practices (such as organ removal) to appear consistent with traditional norms (such as the prohibition against intentionally causing death; cf. [16]). We aim to explore these questions in future research.

### Moral Concept of Death and the Person-as-Moralist Model

Although the motivated reasoning framework is well-suited for explaining our results, an alternative explanation must also be considered. Veatch has argued [7, 22] that the meaning of the word “dead” has evolved in such a way that it now embodies a moral concept. Although “dead” retains its biological connotation in most contexts, when used in the context of organ donation, “the new and different meaning [has] little to do with biology… The word *dead* has come to mean – for legal, ethical, and public policy purposes – ‘having lost full moral standing as a member of the human community’” ([22], p. 10). Full membership in the human moral community affords certain protections, such as the right not to be killed or to not have one’s organs removed. Therefore, to assert that a person is dead within this context is simply to assert “that it is morally, legally, and socially acceptable to remove organs … and that those who do so are not guilty of murder” ([22], p. 11).

In a similar vein, Knobe has argued that people’s basic competencies in making sense of the world, particularly in making sense of other people and their causal interactions with the world, are suffused with moral considerations [9]. That is, certain key concepts involved in the explanation of behavior, such as intention and causation, are partially moral concepts, rather than value-neutral factual concepts. Importantly, Knobe argues that the role of moral judgments in shaping people’s intuitions about intentionality, causation, and related concepts, should not be considered bias or an interfering factor as on the motivated reasoning model. Rather, those moral judgments are part of the normal and proper functioning of our competencies, and indeed, Knobe writes, “It seems that we are moralizing creatures through and through” ([9], p. 328). Hence he describes his view as the *person-as-moralist* model, in contrast with what he calls the *person-as-scientist* model, according to which these concepts are interpreted as value-neutral, factual concepts.

Knobe’s person-as-moralist model states that moral considerations are properly embedded within the concepts of causation, freedom, intention, and other basic concepts, though not including death. Assuming that it is generalized to include the concept of death much as Veatch has described it, this model would explain our results as follows. In the good condition, participants presumably considered organ removal to be “morally, legally, and socially acceptable” ([22], 11). In so doing, they *thereby* judged that the donor is no longer a full member of the human moral community; therefore, they appropriately judged the donor to be dead, in accordance with Veatch’s proposed new definition. In the bad condition, the surgeon’s reprehensible motives and the lack of consent would lead most people to believe that organ removal is not “morally, legally, and socially acceptable”, hence, most people would not judge that the donor in this specific context is dead. Similar explanations may be offered for the relations between the attitude scales and judgments of death. For example, those participants who had more favorable attitudes toward organ donation, in general, were presumably more likely to consider the specific instance of organ removal to be morally permissible (perhaps even in the bad condition), and hence, thereby judged that the donor is dead.

The person-as-moralist model’s explanation of our results is very different than the motivated reasoning model, which posits a reasoning process that biases participants’ judgments toward validating a moral evaluation they have already made. In essence, the motivated reasoning model posits a performance error, whereas on the person-as-moralist model, our participants were not engaged in biased processing at all, but were correctly performing basic competencies and correctly applying core concepts. The results of our studies extend the application of the motivated reasoning and the person-as-moralist models into a new domain, that of judgments about death, but it is not possible to tease apart the two explanations from our experimental evidence alone. Further theoretical and empirical research will be needed to distinguish the explanatory power of the two models.

Although this is an important theoretical question for moral psychology, it is essential to understand that regardless of whether the best explanation is the motivated reasoning or person-as-moralist framework (or some combination of them), on both theoretical frameworks, *moral evaluations of organ transplantation influence judgments of death*. This has significant implications for the bioethical debate about death and organ transplantation.

### Death and Organ Transplantation

According to the dominant interpretation of these issues, neurological criteria for death do not reflect a social construction or a legal fiction designed for the purpose of facilitating organ transplantation. Rather, the determination of death by neurological criteria is grounded in sound biomedical science and philosophical reasoning, and is justified independently of ethical concerns about organ transplantation [2, 3, 23]. Because brain dead donors are in fact *dead* – as a matter of biology – it follows that the removal of organs while the donor remains on the ventilator and with a spontaneously beating heart does not violate the dead donor rule.

However, this interpretation is inconsistent with a body of empirical evidence showing that patients meeting diagnostic standards for brain death retain the capacity for the integrated functioning of the organism as a whole in its maintenance of physiologic stability and resistance of entropy; thus, they are biologically alive [5, 6, 24]. These functions include gas exchange at the alveoli, cellular respiration, nutrition, wound healing, febrile responses to infection, hypertensive, tachycardic, and endocrine responses to incision, neurohormonal regulation of free water homeostasis, growth and sexual maturation in children, and the gestation of healthy fetuses in pregnant women [5, 6]. Rather than being a scientifically justified claim about biology, several scholars have argued that the brain death doctrine embodies or reflects moral evaluations, in particular, that it is permissible to remove vital organs from patients in this devastating condition [7, 8].

Our results provide experimental evidence in support of the assertion that moral evaluations of organ transplantation influence judgments about death and causation, casting doubt on the psychological legitimacy of the mainstream view which holds that neurological criteria for death are justified independently of ethical concerns about organ transplantation. Indeed, there is a long-standing criticism that criteria for death have been redefined in order to allow vital organ procurement to appear consistent with the dead donor rule (cf. [5, 25]). Our experimental results support this interpretation of the literature: Rather than concluding that organ removal is permissible because the donor is dead, people may believe that the donor is dead because they believe organ removal to be permissible.

On the motivated reasoning model, the influence of moral evaluations on judgments of death is an error in reasoning (something like a reverse naturalistic fallacy), whereas on the person-as-moralist model, the embedding of moral evaluations within judgments of death does not reflect an error, since death and causation are partly moral concepts. However, both explanatory models reinforce an important challenge to the mainstream view; namely, scientific or technical expertise grants no particular epistemic, moral, or democratic authority in resolving moral questions [24]. Regardless of whether moral evaluations are appropriately embedded within the concept of death, or if current neurological criteria for death reflect motivated reasoning in which the concept of death is reinterpreted to allow organ procurement to appear consistent with the dead donor rule, our current policies embody or reflect implicit moral evaluations that are not explicitly acknowledged as such. Instead, brain death is treated as if it were an established “scientific fact.” But in so doing, the underlying moral judgments are hidden from view, precluding explicit moral and democratic discourse regarding *why* the removal of vital organs from patients in this devastating condition is ethically permissible, if it is, and if not, then why not.

### Limitations and Future Directions

Limitations of this research include possible concerns about generalizability of the sample due to use of Mechanical Turk, and an online survey environment that may result in potential problems with participant attention. With respect to the first potential limitation, our samples reflected a broad demographic array by age, ethnicity, education, religious beliefs, and other characteristics. Furthermore, numerous traditional psychological findings regarding judgment and decision making have been replicated on Mechanical Turk, suggesting its reliability and utility as a data source [18]. Regarding attention in an online survey environment, we used attention-checks to control for this factor, however, we cannot rule out whether some participants were multi-tasking and perhaps not completely engaged with the survey.

An additional potential limitation of this research is that the bad versions of the vignettes are unrealistic, which might be perceived to limit the application of the findings. However, the aim was not to describe realistic scenarios in order to gauge people’s opinions about organ donation. Rather, the aim was to manipulate the independent variable of moral valence in order to experimentally test the hypothesis that moral valence influences judgments of death and causation. By manipulating this independent variable while holding all other relevant variables constant (and by replicating the findings with different vignettes), we can conclude that differences in judgments of death and causation were caused by the difference in moral valence across the scenarios. Furthermore, the experimental results must be interpreted in conjunction with the correlational findings in which attitudes toward organ donation and euthanasia independently predicted judgments of death and causation, regardless of experimental condition. When taken together, these results establish the basic effect, that moral evaluations influence and predict judgments of death and causation in an organ procurement scenario. In future research we aim to clarify the parameters that moderate and constrain this basic effect.

Finally, there are several differences between the good and bad versions of the vignettes in both studies which are bound up in what we have collectively described as “moral valence”, such as prejudice, consent, and the surgeon’s moral character. In future research we also aim to clarify the role that these distinct components play in thinking about death and causation in the organ procurement context.

## Conclusion

To our knowledge, the studies reported here are the first to experimentally explore the effects of moral evaluations of organ transplantation on beliefs about death and causation. As predicted, we found that moral evaluations of a specific organ removal scenario, as well as individual differences in attitudes toward organ donation and toward euthanasia, can influence and predict judgments about death and causation.
